# Topology Optimization: A Review for Structural Designs Under Statics Problems

**DOI:** 10.3390/ma17235970

**Published:** 2024-12-06

**Authors:** Tianshu Tang, Leijia Wang, Mingqiao Zhu, Huzhi Zhang, Jiarui Dong, Wenhui Yue, Hui Xia

**Affiliations:** 1 School of Civil Engineering and Hunan Engineering Research Center for Intelligently Prefabricated Passive House, Hunan University of Science and Technology, Xiangtan 411201, China; 2 School of Mechanical Engineering, Hunan University of Science and Technology, Xiangtan 411201, China

**Keywords:** topology optimization, structural design, nonlinear theory, linear elasticity, sensitivity analysis

## Abstract

Topology optimization is a powerful structural design method that determines the optimal configuration by distributing materials efficiently within a given design domain while satisfying specified load, performance, and volume constraints. Unlike size and shape optimization, topology optimization is independent of the initial design, offering a broader design space. This paper provides a systematic review of topology optimization methods, covering two theoretical frameworks: linear elasticity and nonlinear theory. Specifically, the review focuses on sensitivity analysis, optimization criteria, and topology solution smoothing within the context of linear elasticity. In the context of nonlinear theory, the review primarily addresses nonlinear phenomena arising from stress constraints, geometric, material, and contact nonlinearities. The paper concludes by summarizing the current state of the field, identifying limitations in existing methods, and suggesting directions for future research.

## 1. Introduction

**Figure 1:**
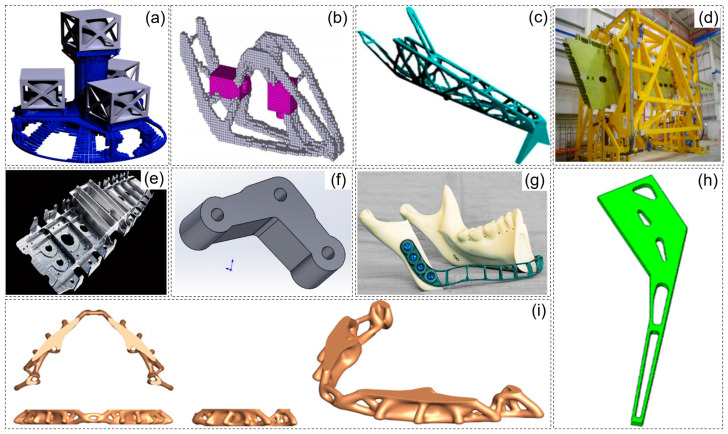
Representative engineering applications utilizing topology optimization design methods: (**a**) satellite support system [2]; (**b**) multi-component layout design for a helicopter pylon [2]; (**c**) topology optimization design of aircraft pylon [2]; (**d**) a typical assembly jig for an aircraft wing [2]; (**e**) aircraft wing design [2]; (**f**) design of subway air conditioning suspension [3]; (**g**) topology optimization of a mandibular reconstruction plate [4]; (**h**) topological optimization of hip spacer reinforcement [5]; (**i**) design of dental framework using topology optimization [6].

Structural lightweight design in machinery and equipment manufacturing is not only a primary means of improving structural performance but also an effective way to reduce energy consumption and emissions. Thus, the development of advanced structural design theories and methods in mechanical engineering provides theoretical and scientific support for advanced functional materials and high-end equipment development. Topology optimization is a powerful structural design method that achieves optimal structural configuration by reasonably distributing materials within the design domain while meeting specified load, performance, and volume constraints. Compared to size and shape optimization, topology optimization is independent of the initial design and offers a broader design space in fields such as automotive, marine, robotics, bioengineering, aerospace, and civil engineering [1,2,3,4,5,6], as shown in Figure 1a–i.

In recent decades, topology optimization has achieved significant theoretical innovations and interdisciplinary integration in mathematics, mechanics, multi-physics fields, and computer science while also playing a growing role in manufacturing and micro- and nanotechnology. However, as topology optimization techniques evolve, the environment it needs to adapt to has become increasingly complex. The limitations of various methods have gained attention from researchers and designers, including low computational efficiency, the need for numerous initial parameters to complete optimal design, poor generality, and the limited versatility of sensitivity calculation methods across different fields. While solving the above problems, the modern optimization algorithms also needs to focus on expanding its basic and applied research in the field of stress and nonlinear optimization [7,8].

**Figure 2:**
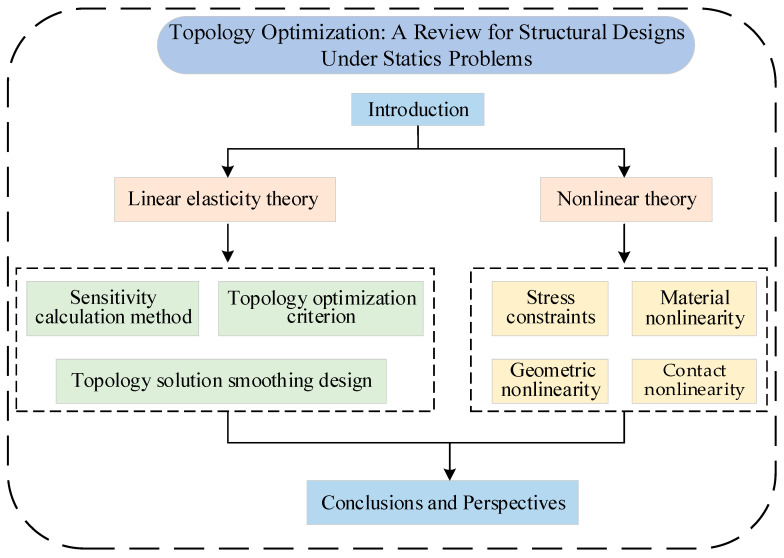
Research framework of this paper.

This paper presents a review of topology optimization methods based on two types of theories: linear elasticity and nonlinearity. The linear elasticity theory primarily focuses on three aspects: sensitivity calculation methods, optimization criteria, and topology solution smoothing design, while the nonlinear theory mainly reviews the nonlinearity caused by stress constraints, geometric nonlinearity, material nonlinearity, and contact nonlinearity. This paper will conclude by enumerating some contemporary issues in topology optimization based on existing research and findings, and it will offer viewpoints and future prospects. It is worth noting that this paper primarily focuses on a review of structural topology optimization design under static problems, emphasizing theoretical analysis and application summaries of topology optimization methods, without addressing specific optimization problems of topology optimization design. The research framework of this paper is shown in Figure 2.

## 2. Topology Optimization Method Under Linear Elasticity Theory

The origins of structural topology optimization can be traced back to Michell’s 1904 criterion for the optimal design of truss structures with the minimal mass under a single condition and stress constraints [9]. Subsequently, scholars like Prager and Rozvany expanded Michell’s theory from trusses to beam structures, thereby introducing the inaugural optimal design theory for topology optimization [10,11]. In recent decades, topology optimization methods and applications have rapidly developed, leading to the establishment of a more complete optimization analysis system.

**Figure 3:**
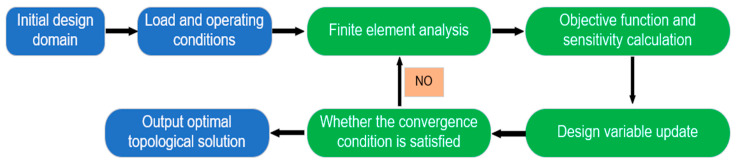
The steps of the structural topology optimization design.

The primary goal of modern topology optimization methods is to identify and judge the optimal material distribution law in the design domain relative to its loads, boundaries, and constraints and integrate computer-aided design (CAD) technology to complete the configuration design of topology decomposition. There are some main steps to complete the optimal design of the structural topology, as shown in Figure 3. First, the initial design domain is established for the specific problem, which is difficult to computerize because the process involves judging the simplified form of the structure in a qualitative way. Second, finite element analysis and sensitivity calculations for topology optimization are performed on the structure. Third, the topology update is completed according to the optimization criteria, and the optimization stop condition is judged. The final step is to output the topological optimal solution that satisfies the design objectives and constraints. It can be seen from the steps of topology optimization analysis that the core problem to complete the topology optimization design of the structure is to set a reasonable sensitivity calculation method and optimization criteria. Therefore, researchers have conducted studies on these two issues [12,13,14,15,16].

Topology optimization methods have developed into the present, since almost all methods are based on completing the structural flexibility minimization design under linear theory, the topology optimization methods for the design of continuum structure flexibility minimization under linear theory have formed a set of more complete theory and validation frameworks. However, with the improvement in topology optimization methods, the upgrading of computer technology, and the development of additive manufacturing technology, topology optimization methods are no longer limited to the initial design of the structure. They now aim to directly convert the optimal topological solution into a product design that can be manufactured using additive manufacturing technology. As a result, topological solutions with clear and smooth boundaries can not only simplify the design process but also enhance their manufacturability.

### 2.1. Sensitivity Calculation Method

Sensitivity calculation refers to the mathematical process of evaluating the extent to which small changes in design variables affect the objective function or constraints. It is a key step in optimization design, providing the direction and magnitude for design updates. Sensitivity results are typically expressed in the form of gradients or derivatives, guiding optimization algorithms toward the optimal solution. Sensitivity calculation methods are at the core of the optimization process, enabling rapid evaluation of the impact of design variables on the objective function.

In response to the different design approaches of various topology optimization methods, their sensitivity calculation methods can be divided into element-based methods, discrete methods, and combined methods.

(1) Element-based methods

Element-based methods [17] generally discretize the problem domain in the finite element, so the finite element solution in the design domain is known or can be approximated. In this class of methods, it is a prerequisite for the finite element method (FEM) to define the geometry of the structure in terms of discrete elements and element nodes that have known degrees of freedom, such as load, temperature, and displacement. The degrees of freedom are transferred between elements through nodes, and numerical equations are established. Finally, these equations are solved to explain the system transformation information in the design domain. Consequently, topological optimization can utilize the degree of freedom of discrete elements in finite element technology to perform sensitivity calculation. The finite element calculation model serves as the geometric model for the optimal topological solution. The most typical design of this class of methods is to complete the geometric modeling by constantly adjusting the material density of the discrete elements, of which the most widely used are methods based on density, topological derivatives, level sets, and phase fields.

Density-based approaches address basic topology optimization problems by discretizing the design domain with solid elements or nodes. One of the most widely used and mathematically well-defined is the solid isotropic microstructure with penalization (SIMP) [18]. Other density-based approaches include the rational approximation of material properties (RAMP), the optimal microstructure with penalization (OMP), the non-optimal microstructures (NOM), and the dual discrete programming (DDP) [19,20,21].

Eschenauer et al. [22] proposed a method for topological derivatives, which is also known as the “bubbling method”. It predicts the influencing derivatives by introducing a microscopic bubble with a center and radius within the design domain, which can guide the ideal placement of the new hole. This method is a special case of the homogenization method [23], where the topological derivative shows holes when the limit of the density changes to 0, which can be used with the level set method or directly for the element-based update method [22,24,25].

Level set methods (LSMs) [26] have the flexibility to handle complex topological boundary variations due to the application of their implicit moving boundary model (IMB) [27]. In traditional LSM, the structure’s boundary is defined by the zero-potential surface of the level set function. The zero-potential surface is derived from the objective function (such as deformation energy, stress, etc.), and the optimal structural configuration can be obtained through the motion and merger of its external boundary [20].

Phase field-based methods [28] correspond to density methods with explicit penalties and regularization. This method was first proposed by Bourdin and Chambolle in order to perform boundary constraints and represent the surface dynamics of phase transition phenomena. It is based on the continuous density field acting directly on the density variables, thereby eliminating the penalty between element interfaces [29].

(2) Discrete methods

The discrete method [30] directly transforms the sensitivity formula into discrete variables related to the cell state, so the numerical calculation method of the sensitivity analysis for the cell is the key to determine whether the method can successfully realize the topology optimization calculation. This type of optimization method faces challenges, such as a small calculation scale and difficulty in describing structural configurations during actual calculations. The most widely used methods are the evolutionary structural optimization method(ESO) [31], the additive evolutionary structural optimization method (AESO) [32], and the bidirectional evolutionary structural optimization method (BESO) [33].

**Figure 4:**
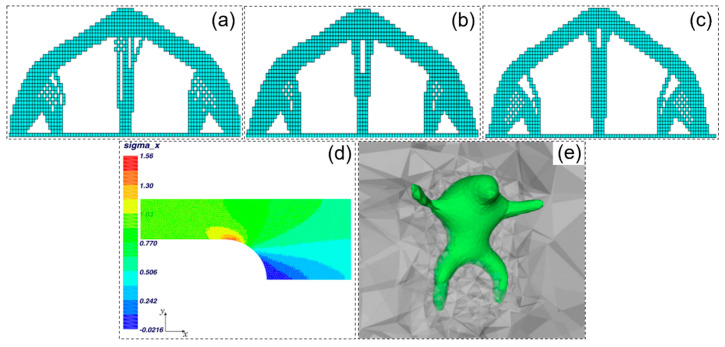
Topology optimization design model obtained by different sensitivity calculation methods: (**a**) ESO [41]; (**b**) WESO [41]; (**c**) BESO [41]; (**d**) X−FEM analysis of a 2D filet 1/4 model in tension [42]; (**e**) smooth model of the 3D arm using DSC [50].

As a heuristic topology optimization method, the ESO adopts the natural principle of “the strong survive, the weak die” in nature and gradually add “useful materials” and remove “useless materials” to drive the structure to achieve optimal topology design. It is found that, because ESO and AESO can only delete or add materials in one direction, it is easy to cause the optimization results to fall into the dilemma of local optimal solutions. BESO was developed to solve this problem, and the effectiveness and efficiency of the method are demonstrated. With the continuous improvement in the stability of the BESO method, it has been applied to the structural design of multiphase materials [34], structural dynamic optimization design [35], and functional gradient porous microstructure design [36,37,38,39,40]. The windowed evolution structural optimization (WESO) method [41], as a branch of the ESO algorithm, retains the design idea of ESO. Although it significantly improves the optimization efficiency by improving the optimization update method, it also has the shortcomings of ESO methods, as shown in Figure 4a–c.

(3) Combinatorial optimization methods

Most sensitivity calculation methods use only the size and shape of cells, nodes, or structures as optimization parameters, while there are not many topology optimization methods that solve the problem in a holistic manner. To address these issues, combinatorial methods have been developed, with the two most representative being the extended finite element method (X-FEM) [42] and the deformable simplicial complex method (DSC) [43], as shown in Figure 4d.

The basic idea of X-FEM is to use unified segmentation to add discontinuous functions to the finite element approximation by introducing generalized and adaptive finite element calculation methods. It is closely related to generalized finite element methods, and both belong to the classification of unified methods [44]. It not only realizes the topology optimization calculation but also completes the output of smooth topology solution. X-FEM can efficiently handle difficult problems involving discontinuities and propagation. To date, the X-FEM method has been primarily developed for crack growth problems, but Belytschko et al. explored the level set description of the X-FEM method on topological optimization problems [45,46,47].

The DSC method is a combination of non-parametric shape optimization methods with the ability to add or remove holes [43]. This method explicitly represents a graph as a piecewise linear curve or surface, which is part of the spatial discretization unit, so that the graph can be retrieved as a set of faces and separates triangles or tetrahedra labeled as interior from those labeled as exterior. This representation allows robust topological adaptation and is only slightly affected by numerical diffusion, due to the explicit representation of the interface. The DSC method is implemented in 2D and 3D and applied to fluid simulation [48,49], topology optimization [50], and cut site computation on Riemannian manifolds [51], as shown in Figure 4e.

### 2.2. Topology Optimization Criterion

The topology optimization criterion refers to the mathematical rules or algorithms that guide the update of design variables during the topology optimization process. It determines the optimization direction and magnitude through sensitivity analysis or optimization theory to achieve the optimal objective function (e.g., structural stiffness and mass) while meeting constraints (e.g., volume and stress). The topology optimization criterion is the core of the optimization process and the key driver of topology updates, directly influencing the design convergence speed and final structural performance.

The optimality criterion methods that support topology optimization design theory can be categorized into gradient-based algorithms and non-gradient-based algorithms. The most representative gradient-based algorithms include the optimality criterion method, convex linearization method, and method of moving asymptotes (MMA). The most representative non-gradient-based algorithms include continuous linear programming, continuous quadratic programming, and genetic algorithm. The following sections describe these typical optimization criteria in detail.

Optimality criterion algorithm (OC) is the most basic gradient-based mathematical method. In this approach, 99 lines of MATLAB with a proportional dependence between the design variables and the value of the objective function [52,53], taking the compliance minimization design problem of Messerschmitt–Bolkow–Blohm (MBB) beam as an example. The process of topology optimization calculation based on OC, nested linear finite element analysis, and design formula is described completely, which lays the foundation for the design of the topology optimization program.

Convex linearization (CONLIN) is a linear mathematical programming method introduced by Fleury and Braidbant [54], which is used for structural optimization criterion that mix variables and take into account the characteristics of the problem. Subsequently, Svanberg [55] proposed the method of moving asymptotes (MMA), which is an improved version based on CONLIN’s method by limiting the movement. MMA not only creates a large number of improved feasible solutions for the optimized problem but also can achieve better design results when dealing with general nonlinear problems considering constraints, design variables, and characteristics of structural optimization problems. This criterion is not only the basis of the homogenization method [23] but also the predecessor of the SIMP method [56,57] (solid isotropic material with penalization, SIMP).

The successive linear programming (SLP) and the successive quadratic programming (SQP) both transform the design variable from a nonlinear problem to a linear problem, and the movable boundary is restricted to a finite region and optimized [58]. Based on the principle of natural selection, natural genetic mechanism, and adaptive search genetic algorithm [59], mainly with the help of the law of “survival of the fittest” in biological evolution, imitating the genetic reproduction mechanism in the process of biological evolution, the coded optimization problem is divided into combinations, and the combinations containing the optimal solutions are searched through iteration. In recent years, it has been well applied in combination with machine learning methods [60].

As shown in Table 1, in order to clarify the differences between each method, we summarize the main advantages and limitations of each topology optimization method; detail its advantages in terms of computational efficiency, solution accuracy, and scope of application; and point out possible limitations such as numerical instability, convergence difficulties, or sensitivity to initial conditions.

### 2.3. Topology Solution Smoothing Design

Despite the significant success of topology optimization in industrial applications, the application of shape optimization in industrial environments is more difficult. One of the main advantages of the optimal material distribution formulation is its ability to work on fixed regular meshes, while shape optimization has to work in the opposite direction. To obtain a smooth topology design, mesh deformation, mesh reconstruction, and finite element error-related difficulties need to be considered. Therefore, methods to generate smooth boundaries based on structural topological solutions with element density have attracted the attention of researchers. Maute et al. [61] originally proposed a method based on mesh adaptation techniques that can not only provide a smooth topology design but also reduce the number of design variables, which was applied to the topology design of shells and elastic plastic structures. Nguyen et al. [62] and Yoo et al. [63] proposed a multi-resolution topology optimization (MTOP) method, which can obtain high-resolution and smooth designs using three different meshes, respectively: displacement, density, and design variable grid. LSM is favored by designers for its clear and smooth topological solutions [64,65]. However, the high complexity and computational inefficiency of these methods limit their attractiveness to both designers and academics.

**Figure 5:**
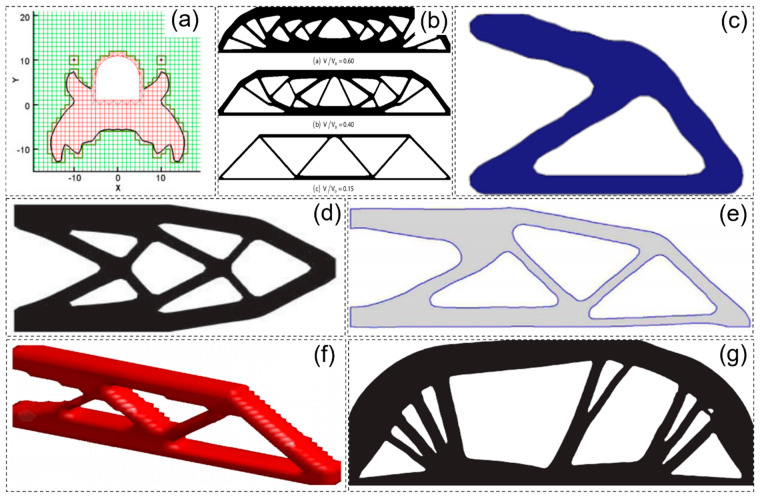
Smooth topology solutions of topology optimization design models obtained by different methods: (**a**) model obtained by the FG BESO method [66]; (**b**) the ITD algorithm obtains the topology of the MBB beam [67]; (**c**) smooth structure boundary using the SVM boundary extraction method [72]; (**d**) cantilever topology optimization using the X−FEM and ITD methods [68]; (**e**) the cantilever topology is based on the two−way evolutionary structure optimization method based on finite element, LSM, and NURB [69]; (**f**) ETO design of a 3D cantilever beam with a concentrated force [70]; (**g**) optimal topology design of Charpy using the RTO method [71].

In recent years, smooth topology optimization based on hybrid methods has made steady progress. Liu et al. [66] proposed a fixed-grid bidirectional evolution structural optimization (FG-BESO) to provide smooth structural boundaries for the topology design of underground tunnels, as shown in Figure 5a. Victoria et al. [67] developed 2D and 3D continuous isoline topology design (ITD) methods based on extended ESO (XESO) and fixed-mesh finite element analysis, as shown in Figure 5b. Abdi et al. [68] integrated the X-FEM and ITD methods into an evolutionary optimization approach to create smooth boundaries, as shown in Figure 5d. Ullah and Trevelyan [69] also integrated BEM, BESO, level set methods, and non-uniform rational b-spline (NURBS) curves to obtain topologies with smooth boundaries, as shown in Figure 5e. Da et al. [70] proposed evolution topology optimization (ETO), which creates the level set function based on the sensitivity of the nodes, as shown in Figure 5f. Martinez-Frutos and Herrero-Perez [71] proposed a robust topology optimization (RTO) method, which replaces the fixed mesh with an adaptive anisotropic sparse mesh and generates a smooth boundary equivalent to the optimal criterion domain, as shown in Figure 5g. Xiao et al. [72] developed a new hybrid approach that combines SIMP and discrete level set methods with a support vector machine (SVM) to achieve smooth boundary design, as shown in Figure 5c. Huang et al. [73,74,75,76] create smooth boundaries based on a fixed-mesh finite element method (FEM) and an evolutionary process that has been applied to 2D and 3D designs. Wang et al. [77] developed an adaptive mesh adjustment method based on SIMP to describe irregular topological boundaries containing isoperimetric elements. However, the hybrid method considers the material interpolation density as a design variable, so the accuracy of the topological solution is related to the material density. The application of adaptive grid or the parametric level set method is limited due to its complexity and poor compatibility. Figure 5 shows the smooth topology solutions of the topology optimization design model obtained by different methods.

## 3. Topology Optimization Method Under Nonlinear Theory

**Figure 6:**
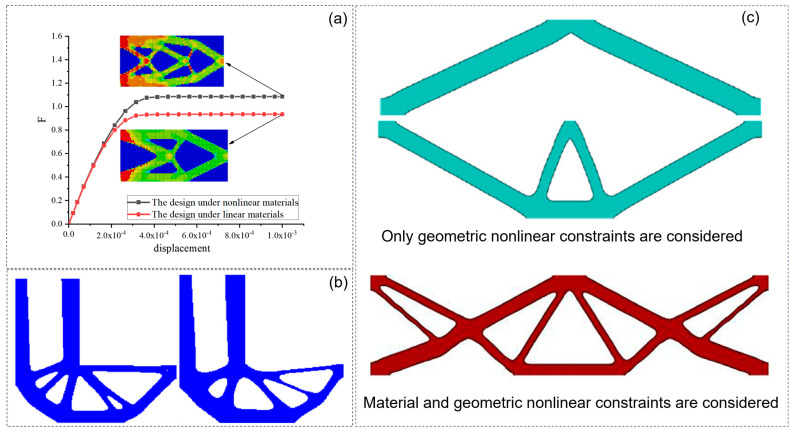
Comparison of topology optimization solutions considering linear and nonlinear conditions: (**a**) material nonlinear constraint [81]; (**b**) stress constraint [82]; (**c**) geometric nonlinear constraint [83,84].

The previously mentioned topology optimization methods show superior performance under linear conditions. However, material and geometric nonlinearities are often neglected in such modeling, and there are no nonlinear problems related to stress constraints. Linear theory is established under the assumptions of continuity, small deformation, uniformity, linear elastic variation of materials, and geometry. Although these assumptions can be effectively applied to most structural designs, nonlinear modeling is required in some cases, such as considering linear elastic materials with yield stress or considering structural energy dissipation under a compliance minimization design. They can be classified as nonlinear structural designs [78,79,80]. Figure 6a shows the differences in displacement, deformation, deflection, and structural morphology of the topological optimal solution designed under linear and nonlinear conditions [81]. The comparison of topology optimization solutions considering linear and other nonlinear conditions is shown in Figure 6b,c [82,83,84]. Considering the sources of nonlinearity in topology optimization, this section reviews the relevant contents of nonlinearity caused by stress constraints, geometric nonlinearity, material nonlinearity, and contact nonlinearity.

### 3.1. Topology Optimization Under Stress Constraints

Structural fracture and fatigue damage caused by stress concentration or high stress value seriously affect the service life of the structure, so it is of great significance to study the topology optimization problem with stress constraints. For stress-constrained optimization problems, Deaton et al. [85] pointed out that there are three challenging problems associated with such problems, which are stress singularity phenomenon, locality of stress constraints, and highly nonlinear stress behavior.

The stress singularity phenomenon primarily appears in density-based optimization methods. The reason is that, when the topological design variables tend to the critical value, the discontinuity of the local stress constraint prevents the optimization algorithm from finding the true optimal solution [86]. To address this issue, researchers have proposed some relaxation methods, such as ε-relaxation [87,88], q p relaxation [89], and stress penalty [90].

The locality of stress constraints will result in a huge computational burden for a sensitivity analysis. Therefore, researchers have proposed stress aggregation functions to reduce the computational burden [91,92]. In these methods, local stress constraints are usually converted into stress condensation formulas, such as the P-norm method or the Kreisselmeier–Steinhauser (K–S) stress measurement method. Although this global stress condensation method improves the computational efficiency, its method is less general. Consequently, researchers have proposed some regional or categorical aggregation techniques to achieve a trade-off between global stress constraint and local stress constraint methods [90,93,94,95].

The stress constraints are highly nonlinear, and the stress state is greatly affected by density variations and local geometric properties, especially in structural boundaries with high curvature, where large stress gradients exist. Therefore, the stress concentration region should be sufficiently captured and subsequently processed by appropriate optimization formulation and solution algorithm, which is very important to overcome the convergence difficulties of topology optimization [90,96,97]. With the increasing research on stress-based topology optimization, the understanding of the problem has become more and more in-depth, and remarkable progress has been made in recent years [85]. Among these studies, some works focused on handling local stress constraints by introducing efficient schemes to avoid possible stress concentration and to relax high stress levels [94,98]. Although the traditional P-norm or K–S aggregation method has high solving efficiency, it is difficult to implement the equivalent constraint on the maximum stress of the structure. As a result, it usually leads to poor local control of the stress distribution or severe violations of certain stress constraints.

**Figure 7:**
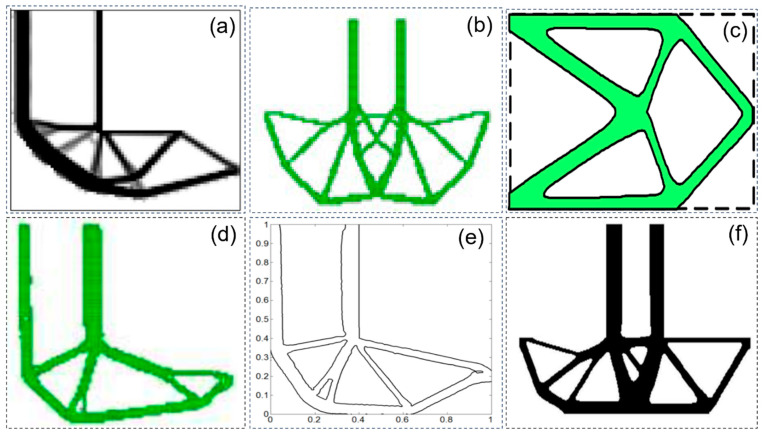
Topology optimization model for different structures under stress constraints: (**a**) topology optimization of a L-shaped structure [99]; (**b**) structural design of T-beams under two load cases [110]; (**c**) optimal design of the short cantilever beam problem [104]; (**d**) topological optimization of stress-constrained continuum L-shape beam structures based on the active constraint technique [110]; (**e**) design of minimum strain energy for a stress-constrained L-beam [106]; (**f**) the design result of a double L-bracket with two stress constraints [111].

Most topology optimization problems that consider stress dependence are based on the SIMP method [93,99,100,101,102] and level set method [97,103,104,105], as shown in Figure 7a–c. Additionally, Cai et al. [106] proposed a new framework for solving stress-constrained topological optimization problems by combining the level set function with the finite cell method, as shown in Figure 7e. Sui Yun kang et al. [107,108,109] proposed a series of strategies to effectively solve stress-constrained topology optimization problems based on the ICM method. Rong Jianhua [110] employed the active constraint technology, combined with the trust domain scheme and the local method, to solve the stress concentration and singularity phenomenon, which further improves the stability and convergence of the optimization process, as shown in Figure 7d. The ESO method does not solve the structural topology optimization with respect to stress minimization using a density-based approach, and the singularity problem can naturally be avoided. Consequently, the stress optimization problem is only concerned with solving the local nature and highly nonlinear behavior of the stress. Fan Zhao et al. [111] extended the bidirectional evolutionary structural optimization (BESO) method to achieve compliance minimization design while satisfying the constraints of volume fraction and maximum von Mises stress. Specifically, the p-norm global stress measurement is introduced to approximate the maximum stress and enhance the traditional design objective to achieve effective control of stress, which not only improves the design flexibility but also ensures that the design can achieve a balance between compliance and stress by introducing Lagrange multipliers, as shown in Figure 7f. However, the problems of unsmooth topological solution boundaries, grid dependence, and numerical instability in the stress optimization problem limit its further development. Thus, the topology optimization problem based on stress optimization needs to be further improved.

### 3.2. Topology Optimization Under Material Nonlinearity

In the optimization theory considering nonlinear behavior of materials, except material nonlinearity, other assumed conditions are basically consistent with linear optimization. Even so, in the material nonlinear analysis, although the stress–strain relationship of the material no longer shows a linear change trend, it is also assumed that the material properties are approximately elastic before the final stress of the material reaches the yield limit—that is, there is no residual deformation when the structure is unloaded.

**Figure 8:**
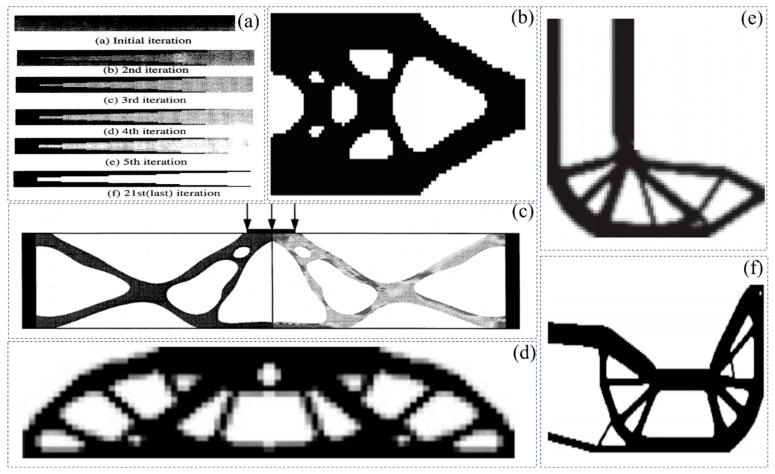
Topology optimization model of different structures under material nonlinearity: (**a**) optimal design of a cantilever subjected to plastic deformation [112]; (**b**) plastic designs of the cantilever [120]; (**c**) optimization result for elastoplastic material and von Mises stresses [114]; (**d**) optimized layout for the multilinear elastic material modeled by ECP [115]; (**e**) material distribution of the optimized U-bracket [117]; (**f**) topology optimization of a U-bracket [124].

In the design of topology optimization under the nonlinearity of materials, the goal of most inelastic topology optimization studies is to maximize the energy absorption methods due to the dependence of the sensitivity on the material path. Among them, Yuge and Kikuchi [112] proposed an optimization method for plastic deformation frame structures, as shown in Figure 8a. Swan and Kosaka [113] proposed the sensitivity analysis (DSA) algorithm for incremental topology design of an energy-type functional by studying structural topology optimization problems involving nonlinear material behavior. Maute et al. [61] extended adaptive material topology optimization to elastic–plastic structures. Schwarz et al. [114] used an elastoplastic material model with respect to equivalent stresses and strains, as shown in Figure 8c. Yoon and Kim [115] realized the topological optimization of nonlinear continuum structures of materials parameterized by element connectivity, as shown in Figure 8d. Bogomolny and Amir [116] proposed an optimization design model for reinforced concrete structures based on topology optimization and elastoplastic material modeling. James and Waisman [117] used a coupled nonlinear continuum damage model to mitigate losses in the optimal topology design, as shown in Figure 8e. Kato et al. [13] investigated the sensitivity of topological optimization analysis considering elastic–plastic deformation and its path dependence. Nakshatrala and Tortorelli [118] developed a topology optimization framework for energy management in dynamically loaded structures with rate-independent elastic–plastic materials. Wallin et al. [119] extended infinitesimal plastic topology optimization to finite strains. Xia et al. [120] improved the stability and effectiveness of the BESO optimization process in nonlinear optimization by introducing the sensitivity dynamic adjustment method and gradually reducing the sensitivity filtering radius, as shown in Figure 8b. Alberdi and Khandelwal [121] proposed a density-based topology optimization framework for the design of energy-absorbing structures with pressure dependent yield behavior. In addition to considering the nonlinear problem of the material itself, the study of Zhao et al. [122,123] also considered the combination of material nonlinearity and stress constraint conditions.

Energy absorption and stiffness are rarely optimized together, because they are closely related—that is, high initial stiffness usually brings relatively high energy absorption. However, both factors need to be considered when optimizing the structure to achieve the maximum stiffness constrained by plastic energy dissipation. To solve this problem, Amir [124] proposed an elastic–plastic topology optimization algorithm to constrain the total plastic deformation while maximizing the stiffness, as shown in Figure 8f. This optimization method is presented as an alternative to stress-based optimization. Although it is more computationally demanding than the theoretical framework of linear elasticity constrained by equivalent stresses, it limits the plastic strain close to zero, thus ensuring that the maximum stress is close to the yield stress.

### 3.3. Topology Optimization Under Geometric Nonlinearity

**Figure 9:**
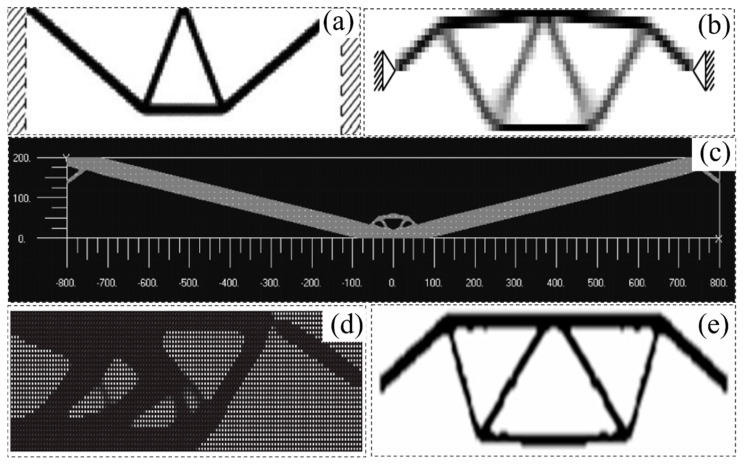
Topology optimization design under geometric nonlinearity: (**a**) optimized topology for large displacement modeling [127]; (**b**) optimal topology resulting from geometrically nonlinear finite element analysis [128]; (**c**) topological optimization under the geometric nonlinearity of the slender beams [129]; (**d**) optimization of the geometric nonlinearity of a cantilever beam [133]; (**e**) optimization of the geometric nonlinearity of a simply supported beam [138].

The initial research to explain geometric nonlinear behavior in topology began in the last two decades. Jog [125] and Bruns [126] argued that structures can be affected by large displacements. However, the application examples presented in these studies failed to effectively demonstrate the existence of significant differences between optimal topologies, objective function values, and linear and nonlinear behaviors. Since the article was published, researchers have conducted a series of studies. Among them, Buhl et al. [127] combined the SIMP method with the nonlinear finite element method to solve the topology optimization of geometric nonlinear problems and verified that there is a significant difference between nonlinearity and linear elasticity, as shown in Figure 9a. Gea and Luo [128] proposed a microstructure-based design method, where nonlinear finite elements are used to optimize the structural topology considering geometric nonlinearities, as shown in Figure 9b. Huang and Xie [129] used the BESO method to study geometric nonlinear structures considering force and displacement loads, as shown in Figure 9c. Fernandes et al. [130] proposed a smoothing evolutionary structural optimization (SESO) method based on ESO for the optimization of two-dimensional elastic problems considering geometric nonlinearities. Ha and Cho [131] illustrated examples of engineering applications that combine the level set method with the geometrically nonlinear method and proposed a formulation using hyper-elastic materials and unstructured meshes for geometrically nonlinear structures in the entire Lagrangian framework.

It is worth mentioning that convergence difficulties often occur in the formulation of density-based topology optimization in the case of nonlinear problems. Buhl et al. [127] proposed a relaxation of the convergence criterion to smooth such a problem. Bruns and Tortorelli [126] proposed a strategy for removing and reintroducing low-density finite element analysis for topology optimization of flexible structures and mechanisms. Lahuerta et al. [132] proposed a technique of combining multi-convex constitutive models with relaxation functions to stabilize finite element analysis with excessive distortions. Wang et al. [133] proposed an interpolation scheme for the virtual domain, which uses the small-strain theory to control the low-density region and the large-strain theory for high-density finite element analysis, as shown in Figure 9d. Luo et al. [134] used a technique of adding hyper-elastic materials in the design domain to alleviate the problems of deformation and numerical instability in low- or medium-density regions.

The studies mentioned earlier highlight the complexity of properly handling topology optimization problems in nonlinear behavior. However, a stable and efficient numerical technique may fundamentally solve the problem of numerical instability in the field of nonlinear topology optimization. Therefore, researchers have tried to apply the boundary element method (BEM) and the finite element method (FEM) to the topological optimization analysis of nonlinear problems [135,136,137,138]. Despite the good convergence rate of FEM, special techniques are required to enforce the basic boundary conditions, which can lead to additional computational costs, as shown in Figure 9e. In addition, the isogeometric approach (IGA) also has a broad application space in topology optimization problems, such as approximating the geometric evolution of NURBS basis functions and problem fields [139,140,141,142]. Although IGA accurately approximates the evolution of geometric shapes, the IGABEM scheme requires the deterministic creation of internal cavities during topology optimization. In summary, efficient numerical schemes for topological optimization solutions under nonlinear constraints are still not available in the literature.

### 3.4. Topology Optimization Under Contact Nonlinearity

**Figure 10:**
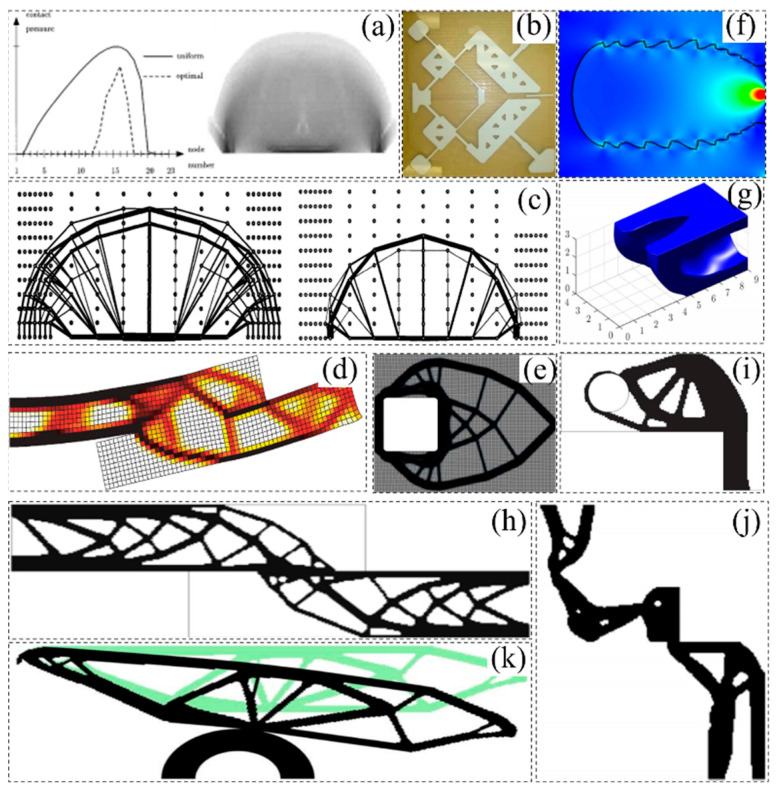
Topology optimization model of different structures under contact nonlinearity: (**a**) the sub-gradient method was used to design the sheet structure [148]; (**b**) design of contact-assisted flexible mechanisms through the use of regularized contact modeling [150]; (**c**) truss optimization by introducing one-way contact conditions [151]; (**d**) global stiffness optimization of frictionless unidirectional contact structures [154]; (**e**) topology optimization to meet manufacturing constraints and one-way contact constraints [155]; (**f**) topological optimization to consider slippage and separation at the contact interface [156]; (**g**) optimization of contact problems under large deformation conditions [159]; (**h**) design of uniformity of the contact pressure [160]; (**i**) stiffness design of frictional contact elastic structures [162]; (**j**) stress-based tribal contact optimization of elastic continuous structures [163]; (**k**) optimization of hyper-elastic structures with large deformation and frictionless contact [164].

Contact nonlinearity is an important problem in topology optimization, which involves the inaccuracy and instability of optimization results caused by the nonlinear behavior of contact surfaces in structural optimization. Consequently, researchers have carried out a series of studies on such problems. Kong and Wang [143] introduced a method based on the boundary element method (BEM) to study the steady-state rolling contact problem of viscoelastic solids. Wriggers [144] summarized several key aspects involved in the early solution of the contact problem, including geometric modeling, interface laws, the development of algorithms, etc. At the same time, a contact geometry model suitable for large deformation was proposed. Liu et al. [145] proposed a finite element algorithm considering contact constraints by directly introducing geometric constraints into algebraic equations, thus effectively solving the contact problem with large deformations and strains. In the contact collision problem, Belytschko and Neal [146] proposed a pinball algorithm for solving the problem, which can be implemented using Lagrange multipliers or penalty methods and does not require iterations to define the contact surface. Refaat and Meguid [147] proposed a new finite element method that can effectively solve the friction contact problem, has better convergence accuracy, can simulate and analyze the friction contact problem more accurately, and is helpful to solve the related problems in engineering applications. Petersson et al. [148] proposed a topology optimization using a sub-gradient method for sheets with surfaces in contact, as shown in Figure 10a. Iban et al. [149] introduced a contact algorithm for nonlinear elastic problems, which is analyzed using the BEM method and is able to handle nonlinear elastic problems, large displacements, and the case of friction forces. Mankame et al. [150] proposed a topology optimization technique for systematically designed contact-assisted variable stiffness mechanisms, which overcomes the difficulties caused by nonlinear contact behavior by using regularized contact modeling, as shown in Figure 10b.

With the development of computer technology, the problem of contact nonlinearity has been paid more and more attention. Some scholars have begun to further study the related problems of topology optimization under contact nonlinearity. Klarbring et al. [151] introduced the unidirectional contact condition into the topology optimization of the truss to optimize the layout of the members of the structure, thereby reducing the weight and cost of the overall structure, as shown in Figure 10c. Myśliński [152] introduced a numerical solution that employs the level set method to optimize the contact problem. Gallego et al. [153] proposed a fast and efficient contact algorithm to solve the contact problem between elastic half-spaces. Desmorat [154] considered the global stiffness maximization problem of frictionless unidirectional contact under the theory of topology optimization and extended the linear elasticity problem to the nonlinear contact problem, as shown in Figure 10d. Stromberg [155] proposed a topology optimization method based on the SIMP method that can simultaneously satisfy manufacturing constraints and unidirectional contact restrictions, as shown in Figure 10e. Additionally, Lawry et al. [156] also introduced a topology optimization method based on the level set method to consider sliding contact and separation on the contact interface to improve the performance and stability of the contact interface, as shown in Figure 10f. Myśliński et al. [157] employed the phase field method to solve the topological optimization problem of an elastic body in unidirectional contact analytically and numerically. Behrou et al. [158] proposed a modified finite element topology optimization method considering the contact and bonding phenomena at the interface and described the geometric material interface by the explicit level set method. Fernandez et al. [159] studied the contact problem of multiple deformable bodies under large deformation conditions in topology optimization and proposed a simulation solution method based on the finite element method to improve the numerical stability, as shown in Figure 10g. Niu et al. [160] proposed an improved method for the topological optimization of elastic continuous structures to improve the uniformity of contact pressure of frictionless contacts in elastic continuous structures, as shown in Figure 10h. Kanno [161] proposed a method to solve the structural topology optimization problem with frictionless unidirectional contact conditions based on Lagrangian duality theory. Niu et al. [162] proposed a topology optimization method for elastic structures considering frictional contacts in 2020, which has been used to design the stiffness maximization of elastic structures with frictional contacts, as shown in Figure 10i. Han et al. [163] proposed a stress-based topology optimization method based on the BESO method and applied it to the frictional contact problem of elastic continuous structures, as shown in Figure 10j. Huang et al. [164] proposed a strength constrained topology optimization method for hyper-elastic structures with frictionless contacts caused by large deformations, as shown in Figure 10k. In the study of contact nonlinear problems, the appearance of self-contact problems will also have an impact on the optimization of structures. Bluhm et al. [165] proposed a method for solving structural topology optimization problems involving self-contact problems by introducing a new medium region as a regularization method for the empty region.

Although researchers have proposed some effective algorithms and methods for topology optimization of contact nonlinear problems, there are still many limitations. These limitations include insufficient guarantees of computational stability and convergence, computational inefficiency, difficulty in constructing accurate nonlinear contact models, and challenges in coupling multi-physics fields. Therefore, future research should focus on developing more efficient and generalized topology optimization algorithms to enhance computational efficiency and accuracy while providing optimized solutions for practical engineering design.

## 4. Conclusions and Perspectives

(1) Traditional optimization methods have complex parameter settings and low computational efficiency. Topology optimization based on functional requirements has been well developed, but as an engineering-oriented design tool, the simplicity and efficiency of the method should be considered. In the modern design method, the continuum topology optimization method based on linear theory, when used in the conceptual design of products, has the characteristics of simple operation and clear topology solution, so it is more favored by designers. Although the SIMP and ESO methods have the advantages of high computational efficiency, easy to learn and use, and easy to integrate, they still have some significant defects, such as difficult to extract topology solutions and great influence of design parameters on the results [1,84,166]. Therefore, how to effectively eliminate the shortcomings of traditional methods while maintaining the advantages of existing optimization methods is an urgent problem to be solved.
(2) Topology optimization methods based on stress constraint problems are numerically unstable. The stress aggregation function based on the maximum stress constraint can significantly reduce the calculation amount of the sensitivity analysis due to the stress constraint. However, due to the highly nonlinear stress constraints and sensitivity to local material changes, not all models can deduce smooth convergence results. Therefore, designing an efficient stress aggregation function, which can effectively deal with the stress-constrained topology optimization problem, is a key scientific problem to further solve the stress-constrained problem.
(3) The structural compliance minimization design problem considering stress constraints is susceptible to the perturbation of weighting factors. In the actual analysis and calculation, the sensitivity value of the element obtained by the stress aggregation function is far less than the compliance value. Therefore, when the stress constraint is insufficient in the optimization, a weight factor with too large a value is inserted suddenly, which will perturb the sensitivity value in the multi-objective constraint and cause the instability of the numerical calculation. In order to obtain the optimal solution, the designer needs to repeatedly adjust the weight factor, which increases the design difficulty and reduces the design efficiency. Therefore, developing a weight factor that can be adjusted adaptively according to the change of stress constraint level is a key technical problem to solve the insufficient stress constraint and the instability of numerical calculation in multi-objective constraints.
(4) There is a lack of systematic research on structural topology optimization design based on nonlinear theory. Although the topology design under nonlinear theory is more in line with engineering practice, there are a few structural topology optimization methods considering nonlinearity at present, and the material nonlinear optimization problem, geometric nonlinear optimization problem, and stress constraint problem are discussed separately, and these problems are simply combined by the weight factor in linear optimization theory. However, using the weight factor as the sensitivity of the bridge to approximate the change in the objective function will lead to problems such as low accuracy of the sensitivity calculation and difficult convergence in the optimization design of complex structures. The difficulty of topology optimization for nonlinear structures is how to obtain the “best” topology design in a robust way while ensuring computational efficiency. Although the solution may not be the theoretical optimal, it meets the optimal structure form under multiple constraint 0 conditions. Therefore, it is necessary to reexamine the sensitivity calculation method under linear theory from the perspective of optimization design theory. On the basis of nonlinear theory and software design, developing a general and efficient topology optimization calculation theory is a key scientific issue to solve the topology optimization design problem of nonlinear structures. With the development of computing technology, for the instability in nonlinear problems, more accurate nonlinear constitutive relations can be adopted; the adaptive mesh refinement technology is introduced to dynamically adjust the mesh density according to the distribution of stress concentration, geometric boundaries, or nonlinear regions; neural networks and machine learning are utilized to accelerate the optimization solution and quickly predict nonlinear behaviors, thereby reducing computing costs; large-scale parallel computing is used to handle complex nonlinear problems, thereby improving the solution efficiency.
(5) There is less discussion on how topology optimization methods can guide the design of engineering structures. Although topology optimization methods have been applied in many industries, there is a lack of discussion on general issues, particularly nonlinear optimization problems. In the optimization, the objective volume constraint is a prerequisite, which is effective for the topology optimization method under linear theory, because it does not consider the problem of insufficient bearing capacity and excessive deformation of the materials that make up the structure. However, this condition is obviously no longer applicable in the structural topology optimization considering nonlinearity, and too small a target volume constraint will lead to the topology optimization not able to find the optimal solution. For the topology optimization design of unknown structures, without any characteristic parameters, designers cannot predict what kind of volume constraints are optimal for the performance of the topology solution. Furthermore, for the large-scale structure composed of a variety of materials in engineering, except for the topology optimization design theory based on composite materials, there is no more simple design idea to guide the topology optimization design of a large-scale structure. Therefore, in order to apply topology optimization methods to structural design, in addition to advanced topology optimization design algorithms, it is necessary to develop efficient and practical structural design schemes, which is a key technical problem to solving the structural lightweight design problems encountered in engineering.

## Figures and Tables

**Table 1:** Advantages and limitations of different topology optimization methods.

Topology Optimization Methods	Advantages	Limitations
**Density methods** [18,19,20,21]	1. Computationally efficient and easy to implement. 2. The solution accuracy is reasonable. 3. Can be used for continuum structures.	1. Produces intermediate density value. 2. The results were sensitive to punishment factors. 3. The results may have “spurious holes”.
**Homogenization methods** [24]	1. Wide application range, can deal with periodic microstructure. 2. Multi-scale optimization can be achieved.	1. Numerical instability. 2. Convergence is difficult and the speed is slow.
**Level set methods** [26]	1. The topological boundary is clearly described. 2. Handle complex boundaries with high accuracy. 3. Suitable for complex geometries and heterogeneous material distributions.	1. High computational cost and low efficiency 2. Sensitive to the initial level set function
**Phase field methods** [28]	1. No explicit boundary description required. 2. High accuracy of topological boundary description.	1. Large amount of calculation and low efficiency 2. Complex parameter selection
**ESO** [41]	1. Applicable to large scale problems. 2. Less dependent on the initial design.	1. Poor convergence and easy to fall into local optimal solutions 2. The solution accuracy may be affected by the material removal strategy
